# Sexual dream and family relationships in frequent sexual dreamers and healthy volunteers

**DOI:** 10.1097/MD.0000000000021981

**Published:** 2020-09-04

**Authors:** Xu Shao, Chu Wang, Yanli Jia, Wei Wang

**Affiliations:** Department of Clinical Psychology and Psychiatry, School of Public Health, Zhejiang University College of Medicine, Hangzhou, Zhejiang, China.

**Keywords:** continuity hypothesis, family relationship, sexual behavior, sexual dream

## Abstract

Early life family relationships affect the sexuality in adulthood, and these influences might be reflected in sexual dreams. The present study was designed to investigate the exact associations between family relationships and sexual dream experience. We therefore invited 62 frequent sexual dreamers (dreamers) and 104 healthy volunteers (controls) to answer the Sexual Dream Experience Questionnaire (SDEQ) and the Family Relationship Questionnaire (FRQ). Compared to controls, dreamers scored higher on all SDEQ factors and sexual dream frequency, higher on FRQ Paternal Abuse, and lower on FRQ General Attachment and Maternal Freedom Release. In controls, Paternal Abuse was associated with Joyfulness, Maternal Dominance with Aversion, and Maternal Abuse with dream frequency (–). In dreamers, Paternal Abuse was associated with Aversion, Bizarreness and dream frequency, and Maternal Freedom Release with Aversion (–). In conclusion, there were pronounced associations between sexual dreams and family relationships in frequent sexual dreamers. Paternal Abuse in particular was associated with sexual dream experience. Adverse family relationships might induce frequent sexual dream occurrence, and family therapy or early intervention of Paternal Abuse might alleviate the negative sexual dream experience.

## 1 Introduction

Sexual dream is one kind of typical dreams described in human. More than 70% of people have experienced sexual dreams in general population.^[1–3]^ According to a Canadian study, sexual experience was the second most-frequent themes in dream, after the one of being chased or pursued.^[1]^ Contents of sexual dreams are mainly sexual intercourse, flirting, kissing or sexual fantasies,^[4,5]^ and negative ones such as aggression-involving and rape.^[6]^

Daily dreams are interpreted as reflections of one's waking states and concerns, and psychological variables according to the continuity hypothesis.^[7,8]^ Dreams did not simply depict waking-life traits, but rather further elaborated the elements formed during waking life.^[9,10]^ Therefore, sexual dreams might manifest an individual's sexuality during waking time in general. Investigators found that waking-life sexual fantasies were direct-positively related to sexual dreams.^[11]^ In a Canadian university student sample, sexual interactions in dreams were positively correlated with sexual fantasy, sexual daydreaming, and orgasmic experience during waking time.^[6]^ In female participants, the realistic romantic problems including jealousy and infidelity were reflected in sexual dreams.^[12]^ In male participants, Yu found that sexual behaviors only predicted part of sexual dream contents, which revealed that sexual dreams compensated for waking-state sexual behaviors.^[13]^ Thus, recent literature and the continuity theory suggest that sexual dreams implicitly reflect people's attitudes towards sexuality, sex-related issues, or waking-state sexual behaviors, and fulfill individual's sexual desires which are not completely satisfied at waking-state.

Attitudes towards sexuality or sexual behaviors are affected by multidimensional factors, such as endocrine hormone,^[14]^ alcohol consumption,^[15]^ physical fitness,^[16]^ mental state,^[17]^ sexual knowledge,^[18,19]^ and religion.^[20]^ Moreover, previous results have indicated that family factors contribute to sexual attitudes or behaviors.^[21,22]^ For instance, familial intactness, close parent–adolescent relationship, and parental monitoring were protective factors from early sexual behaviors.^[23]^ Parental communication about sexual norms of love and respect was negatively associated with the permissive sexual attitudes.^[24]^ In addition, family functions such as the positive affective response, communicating, problem solving, and behavioral controlling between family members were associated with the knowledge and attitude toward sexuality in adolescents.^[25]^ On the contrary, adolescents’ bad attitude toward sexuality was related to the poor interpersonal relationships with family members^[26]^; and individuals’ self esteem and body image could be damaged by paternal/maternal overprotection.^[27]^ Besides, childhood maltreatment was negatively related to women's sexual or relationship satisfaction.^[28]^ Early negative familial experiences were strongly related to many kinds of sexual disorders,^[29]^ and the abnormal parent–child bonding and detachment in early life led to deviant sexual fantasies in sexual offenders.^[30]^ Therefore, parent–child attachment, parental support, and parental encouragement might lead to individuals’ positive sexual experiences in later life, while parental overcontrol or abuse might lead to emotionally negative ones. As daytime activities are reflected in dreams, the sexual dream experience might be affected by family relationships accordingly.

However, up to date, the exact relationship between family factors and sexual dream experience is still unclear. One reason for the literature scarce might be the inadequate quality of sexual dream and family relationship measurements. Batteries such as the Typical Dream Questionnaire^[31]^ and the Dream Content Questionnaire^[32]^ have helped to describe the sexual activities or thoughts in dreams, but they are not comprehensively structure-validated. Comparatively, the Sexual Dream Experience Questionnaire^[33]^ has been developed as a structure-validated measure which covers four scales of Joyfulness, Aversion, Familiarity, and Bizarreness, and a scale measuring sexual dream frequency. As to the family relationship, questionnaires such as the Parental Bonding Instrument (PBI)^[34]^ and the Egna Minnen av. Barndoms Uppfostran (EMBU)^[35]^ were commonly used to measure parental rearing experiences. But another instrument, the Family Relationship Questionnaire (FRQ),^[36]^ is structure-validated and measures both positive and negative influences by either parent. Therefore, SDEQ and FRQ might be used to elucidate the relationships between sexual dream experience and family relationships.

In the present study, we have invited both frequent sexual dreamers and normal healthy volunteers to answer SDEQ and FRQ. We have hypothesized that:

1. frequent sexual dreamers scored significantly higher on SDEQ Joyfulness, lower on SDEQ Aversion or Bizarreness, higher on FRQ General Attachment, Freedom Release or Encouragement, and lower on FRQ Abuse or Dominance; and
2. in frequent sexual dreamers, General Attachment, Freedom Release or Encouragement was associated with Joyfulness, and Abuse or Dominance with Aversion or Bizarreness.

## 2 Materials and methods

### 2.1 Participants

We enrolled 104 healthy participants (controls: 38 women and 66 men; mean age, 22.19 years ± 3.88 SD, age range, 18–35 years) who reported 1 to 3 times of clearly-remembered sexual dreams annually, and 62 frequent sexual dreamers (dreamers: 22 women and 40 men; mean age, 23.19 ± 5.07, age range, 18–39) who reported no less than 3 times of sexual dreams monthly. All participants were recruited from either host university, community, or local psychiatric clinics. No differences of age (*t* = 1.43, *P* = .16, 95% confidence interval [CI]: −0.38–2.38) or gender (*χ*^2^ = 0.02, df = 1, *P* = .89) were found between the two groups. Participants had received basic education and had no difficulty understanding or completing the test. They were also confirmed not to have confounding factors such as schizophrenia, schizoaffective disorder, prior history of head injury, alcohol or tobacco abuse, psychoactive substance abuse, sexual dysfunction, paraphilic disorder, or any other medical condition influencing sexual function via a semi-structured interview by an experienced psychiatrist according to the *Diagnostic and Statistical Manual of Mental Disorders*, 5th ed.^[37]^ In addition, participants were free from drug or alcohol, and from viewing pornographic videos or movies, for at least 72 h prior to the test. The study protocol was approved by the Medical Ethics Committee of School of Public Health, Zhejiang University and all participants gave their written informed consent.

### 2.2 Instruments

Participants were asked to complete the following questionnaires in a quiet room.

1. The Sexual Dream Experience Questionnaire (SDEQ)^[33]^ has one item measuring the sexual dream frequency and 32 items measuring four factors of dream experience (8 items each factor): Joyfulness describing the happiness and satisfaction of sexual dreams, Aversion the guilt, shame, fear, and discomfort towards sexual dreams, Familiarity the normal scenes appeared in sexual dreams, and Bizarreness the unusual behaviors or thoughts in sexual dreams. Participants were asked to rate the items using the Likert type scale (1-very unlike me, 2-moderately unlike me, 3-somewhat unlike and like me, 4-moderately like me, and 5-very like me). The internal alphas of each SDEQ factor in the present study were shown in Table 1.
2. The Family Relationship Questionnaire (FRQ)^[36]^ is designed to evaluate the core features of the experienced family relationships up to 16 years old. It has 43 items including General Attachment (5 items; describing children's affectionate dependence on family members), Paternal/Maternal Encouragement (5 items each; describing the positive incentives, harmonic environment and spiritual/material needs offered by parents), Paternal/Maternal Abuse (5 items each; describing the physical or psychological maltreatment and criticism from parents), Paternal/Maternal Freedom Release (5 items each; describing the autonomy of decision-making or daily behavior given by parents), and Paternal/Maternal Dominance (4 items each; describing the parental authority in family affairs). Each FRQ item was rated using the same 5-point Likert scale as in SDEQ. The internal alphas of each FRQ factor in the present study were also shown in Table 1.

**Table 1:**
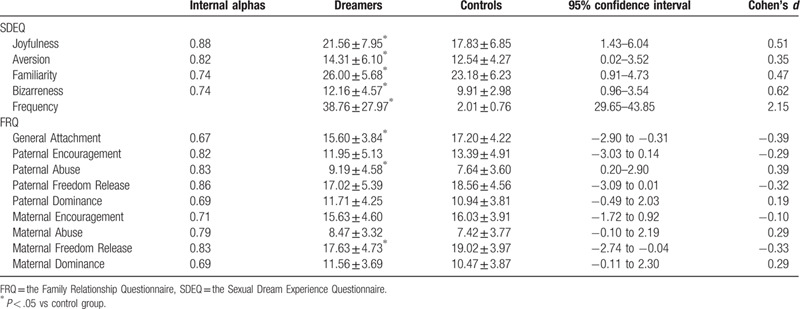
Internal alphas (N = 166) and scale scores (mean ± SD) of the Sexual Dream Experience Questionnaire and the Family Relationship Questionnaire in frequent sexual dreamers (Dreamers, n = 62) and healthy volunteers (Controls, n = 104).

### 2.3 Data analyses and statistics

Data analyses were conducted using Statistical Product and Service Solutions for windows (SPSS, version 19.0, IBM, Armonk, NY). Multivariate analysis of variance (MANOVA) was applied to the 4 SDEQ or 9 FRQ factors in two groups. The independent Student *t* test was also employed to look for the potential group differences. The annual sexual dream frequency was also analyzed by the *t* test. Cohen's d was calculated for each comparison to show the effect size. The partial correlation test of SDEQ (including dream frequency) and FRQ factors was applied in each group. Given the proven effect of age and gender on sexual dreams,^[38,39]^ we controlled age and gender as covariates in the partial correlation test to rule out their possible influences. Moreover, multiple linear regression analysis (stepwise method and adjusted for age and gender) was employed respectively in each group to search for the relationships between SDEQ and FRQ factors, taking FRQ factors as potential predictors for SDEQ ones. A *P* value <.05 was considered to be significant. In order to avoid the chances of Type I error, the absolute value of coefficient larger than 0.25 was considered as significant for correlation, and the absolute beta value larger than 0.25 for prediction.

## 3 Results

There were significant differences on SDEQ scale scores between the two groups (Pillai's Trace = 0.14, Wilks’ λ = 0.86, Hotelling's Trace = 0.16, *F* [4, 161] = 6.43, *P* < .001, partial η^2^ = 0.14). Dreamers scored significantly higher than controls did on the Joyfulness (*t* = 3.20, *P* = .002), Aversion (*t* = 2.01, *P* = .05), Familiarity (*t* = 2.91, *P* = .004), Bizarreness (*t* = 3.46, *P* = .001), and dream frequency (*t* = 10.35, *P* < .001). Meanwhile, there were significant differences of FRQ scores between the two groups (Pillai's Trace = 0.11, Wilks’ λ = 0.89, Hotelling's Trace = 0.12, *F* [9, 156] = 2.15, *P* = .03, partial η^2^ = 0.11). Dreamers scored significantly higher than controls did on the Paternal Abuse (*t* = 2.28, *P* = .03), but lower on General Attachment (*t* = −2.45, *P* = .02) and Maternal Freedom Release (*t* = −2.03, *P* = .04) (Table 1).

In controls, Paternal Abuse (*r* = .27, *P* = .01) was correlated with joyfulness, and maternal dominance (*r* = .27, *P* = .01) with Aversion. In Dreamers, Paternal Abuse (*r* = .28, *P* = .03) and Maternal Freedom Release (*r* = −.32, *P* = .01) were correlated with Aversion, Paternal Abuse (*r* = .46, *P* < .001) with Bizarreness, and Paternal Abuse (*r* = .34, *P* = .01) with dream frequency (Table 2).

**Table 2:**
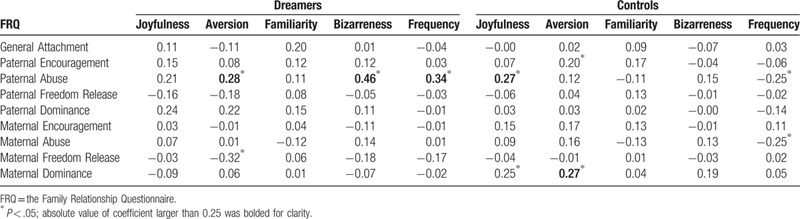
Partial correlation (adjusted for age and gender) between the Sexual Dream Experience Questionnaire and the Family Relationship Questionnaire in frequent sexual dreamers (Dreamers, n = 62) and healthy volunteers (Controls, n = 104).

Considering the prediction of SDEQ factors by the FRQ factors, adjusted *R*^2^ were ranged from 0.07 to 0.22 in controls, and from 0.10 to 0.22 in dreamers. In controls, maternal abuse (β = −0.28; *t* = −2.99, *P* = .003) predicted dream frequency (*F* [1, 102] = 8.94, mean square error (MSE) = 4.76, *P* = .003; adjusted *R*^2^ = 0.07). While in dreamers, Maternal Freedom Release (β = −0.30; *t* = −2.53, *P* = .01) predicted the aversion (*F* [2, 59] = 7.19, MSE = 222.16, *P* = .002; adjusted *R*^2^ = 0.17), Paternal Abuse (β = 0.49; *t* = 4.29, *P* < .001) predicted the Bizarreness (*F* [1, 60] = 18.43, MSE = 299.95, *P* < .001; adjusted *R*^2^ = 0.22), and Paternal Abuse (β = 0.33; *t* = 2.73, *P* = .01) also predicted dream frequency (*F* [1, 60] = 7.45, MSE = 5270.13, *P* = .01; adjusted *R*^2^ = .10) (Table 3).

**Table 3:**
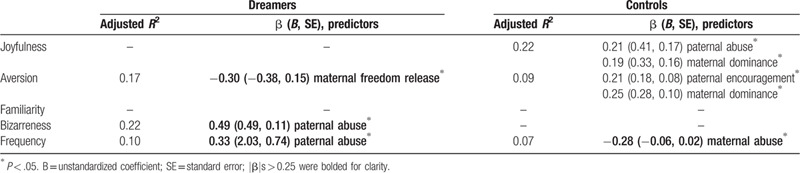
Stepwise multiple linear regression predicting the Sexual Dream Experience Questionnaire factors (including dream frequency) with the Family Relationship Questionnaire factors (adjusted for age and gender) in frequent sexual dreamers (Dreamers, n = 62) and healthy volunteers (Controls, n = 104).

## 4 Discussion

To the best of our knowledge, it is the first comprehensive study addressing associations between family relationships and sexual dream experience in frequent sexual dreamers. Compared to healthy volunteers, frequent sexual dreamers scored higher on all SDEQ factors including dream frequency, higher on FRQ Paternal Abuse, and lower on FRQ General Attachment and Maternal Freedom Release, which partly confirmed our first hypothesis. Paternal Abuse and Maternal Freedom Release significantly predicted some sexual dream experience factors in frequent sexual dreamers, which also partly accorded with our second hypothesis.

Generally, sexual pleasure or emotional satisfaction during sexual dreams was frequently reported by men^[13]^ and women.^[40]^ There are also some cultural-specific differences on sexuality, for instance, East-Asian people tended to have higher sexual guilt than their Western counterparts.^[41,42]^ Therefore, our Chinese dreamers might have averse feelings during waking time due to the conservative sex values despite that they might have pleasant sexual dreams. These explained our results that dreamers scored higher on both Joyfulness and Aversion factors. In addition, dreamers had higher Familiarity, which was in line with that these individuals were familiar with persons involved in sexual dreams.^[6]^ Previous results have also demonstrated that bizarreness enhanced recall of dream contents,^[43]^ which might account for the higher Bizarreness in our dreamers who also had frequent dream recalls.

Dreamers scored lower on General Attachment in our study, which agreed with that individuals with insecure interpersonal attachment were likely to report dreams.^[44]^ Frequent dreamers also had higher Paternal Abuse, which could be explained by that physical abuse by fathers increased adolescents’ level of internalizing behavior problems^[45]^ and that these problems increased dream recalls.^[46]^ Moreover, our dreamers scored lower on Maternal Freedom Release, which might be due to that mothers communicated with children more often than fathers about sex and mothers promoted sexual abstinence.^[47,48]^ While individuals’ idea of sexuality was suppressed, it might be persistent in dreams instead.^[49]^

In our controls, Paternal Abuse was associated with Joyfulness, which might be due to that healthy individuals had positive sex-related self-cognition, and this cognition offset the negative influence of abuse on sexual functioning and satisfaction.^[50]^ Besides, Maternal Dominance was associated with Aversion, which was in line with that maternal psychological control was related to anxiety through adolescent emotional dysregulation,^[51]^ and that anxiety led to negative dream affect.^[52]^ Furthermore, Maternal Abuse was negatively associated with dream frequency, which accorded with that maternal abuse longitudinally predicted adolescents’ depressive symptoms in early adulthood,^[53]^ and that depression was linked with the loss of sexual interest or arousal.^[54]^

In our dreamers, Paternal Abuse was associated with Aversion, Bizarreness, and dream frequency. Previous studies showed that paternal physical abuse predicted adolescent sexual victimization.^[55]^ Sexual abuse increased psychosocial problems^[56]^ and sexual aversion,^[57]^ and victims had increased compulsive sexual activities in spite of their traumatic belief that sex was bad.^[58]^ Based on the continuity hypothesis, these waking-state cognitions might extend to dreams, and our dreamers might recognize their highly-recurrent sexual dreams repulsive and weird. In addition, Maternal Freedom Release was negatively associated with Aversion, which agreed with that adolescents talked about sex more often with their mothers^[48]^ and that the parental support was related to sexual satisfaction.^[59]^

However, there were several design flaws in the present study. First, sample sizes of two groups were relatively small, and the current results need to be replicated with larger samples, especially of frequent sexual dreamers. Secondly, participants’ daytime sexual behaviors or attitudes were not recorded, which might help explain our results. Thirdly, our study design was cross-sectional, and a longitudinal design might further detail the family relationship involvement in sexual dreams. Notwithstanding, we have found the enhanced sexual dream experience and adverse parent–child relationships in frequent sexual dreamers. Especially, Paternal Abuse was a robust predictor of unpleasant but frequent dream experience.

Parent-child issues, especially sex-related ones, in early family life could influence individual's later-life sexuality reflected in sexual dreams. Therefore, family therapy and especially early life intervention of father–child relationship to reduce the negative affect of frequent sexual dreams.

## Acknowledgments

The authors thank Dr Hongying Fan for helping the data-analyses of this study.

## Author contributions

**Conceptualization:** Wei Wang.

**Data curation:** Xu Shao, Chu Wang, Yanli Jia.

**Formal analysis:** Xu Shao.

**Methodology:** Wei Wang.

**Project administration and supervision:** Wei Wang

**Writing – original draft:** Xu Shao, Wei Wang.

**Writing – review & editing:** Xu Shao, Wei Wang.

## References

[1] Nielsen TA Zadra AL Simard V The typical dreams of Canadian university students. Dreaming 2003;13:211 35.

[2] Schredl M Ciric P Götz S Typical dreams: stability and gender differences. J Psychol 2004;138:485 94. 15612605 10.3200/JRLP.138.6.485-494

[3] Yu CKC Typical dreams experienced by Chinese people. Dreaming 2008;18:1 0.

[4] Yu CKC Fu W Sex dreams, wet dreams, and nocturnal emissions. Dreaming 2011;21:197 212.

[5] Zadra AL Sex dreams: what do men and women dream about? Sleep 2007;30:A376.

[6] King DB DeCicco TL Humphreys TP Investigating sexual dream imagery in relation to daytime sexual behaviours and fantasies among Canadian university students. Can J Hum Sex 2009;18:135 46.

[7] Domhoff GW Finding Meaning in Dreams: A Quantitative Approach. New York, NY: Plenum Press; 1996.

[8] Domhoff GW The Scientific Study of Dreams: Neural Networks, Cognitive Development, and Content Analysis. Washington DC: American Psychological Association Press; 2003.

[9] Blagrove M Pace-Schott EF Trait and neurobiological correlates of individual differences in dream recall and dream content. Int Rev Neurobiol 2010;92:155 80. 20870067 10.1016/S0074-7742(10)92008-4

[10] Ferro A Some implications of Bion's thought: the waking dream and narrative derivatives. Int J Psychoanal 2002;83:597 607. 12088558 10.1516/002075702760022569

[11] Schredl M Desch S Röming F Erotic dreams and their relationship to waking-life sexuality. Sexologies 2009;18:38 43.

[12] Clarke J DeCicco TL Navara G An investigation among dreams with sexual imagery, romantic jealousy and relationship satisfaction. Int J Dream Res 2010;3:54 9.

[13] Yu CKC Lust, pornography, and erotic dreams. Dreaming 2013;23:175 93.

[14] Carosa E Lenzi A Jannini EA Thyroid hormone receptors and ligands, tissue distribution and sexual behavior. Mol Cell Endocrinol 2018;467:49 59. 29175529 10.1016/j.mce.2017.11.006

[15] Wells BE Rendina HJ Kelly BC Demographic predictors of event-level associations between alcohol consumption and sexual behavior. J Urban Health 2016;93:155 69. 26678072 10.1007/s11524-015-0015-8 PMC4794469

[16] Jiannine LM An investigation of the relationship between physical fitness, self-concept, and sexual functioning. J Educ Health Promot 2018;7:57. 29922686 10.4103/jehp.jehp_157_17 PMC5963213

[17] Derbyshire KL Grant JE Compulsive sexual behavior: a review of the literature. J Behav Addict 2015;4:37 43. 26014671 10.1556/2006.4.2015.003 PMC4500883

[18] Gaioso VP Villarruel AM Wilson LA A path analysis of Latino parental, teenager and cultural variables in teenagers’ sexual attitudes, norms, self-efficacy, and sexual intentions. Rev Lat Am Enfermagem 2015;23:500 11. 26312635 10.1590/0104-1169.0398.2581 PMC4547074

[19] Hlongwa M Mashamba-Thompson T Makhunga S Evidence on factors influencing contraceptive use and sexual behavior among women in South Africa: a scoping review. Medicine 2020;99:e19490. 32195948 10.1097/MD.0000000000019490 PMC7220276

[20] Ahrold TK Farmer M Trapnell PD The relationship among sexual attitudes, sexual fantasy, and religiosity. Arch Sex Behav 2011;40:619 30. 20364304 10.1007/s10508-010-9621-4 PMC4419361

[21] Ashcraft AM Murray PJ Talking to parents about adolescent sexuality. Pediatr Clin North Am 2017;64:305 20. 28292447 10.1016/j.pcl.2016.11.002 PMC5517036

[22] Pop MV Rusu AS Couple relationship and parent-child relationship quality: factors relevant to parent-child communication on sexuality in Romania. J Clin Med 2019;8:386. 10.3390/jcm8030386 PMC6463176 30893950

[23] Lenciauskiene I Zaborskis A The effects of family structure, parent-child relationship and parental monitoring on early sexual behaviour among adolescents in nine European countries. Scand J Public Health 2008;36:607 18. 18775817 10.1177/1403494807088460

[24] Overbeek G van de Bongardt D Baams L Buffer or brake? The role of sexuality-specific parenting in adolescents’ sexualized media consumption and sexual development. J Youth Adolesc 2018;47:1427 39. 29536329 10.1007/s10964-018-0828-3 PMC6002450

[25] Huerta-Franco R de Leon GD Malacara JM Knowledge and attitudes toward sexuality in adolescents and their association with the family and other factors. Adolescence 1996;31:179 92. 9173784

[26] Ferreira M Nelas P Duarte J Family culture and adolescent sexuality. Aten Primaria 2013;45:216 22. 10.1016/S0212-6567(13)70025-8 PMC8171414 23735567

[27] Bhandari S Winter D Messer D Family characteristics and long-term effects of childhood sexual abuse. Br J Clin Psychol 2011;50:435 51. 22003952 10.1111/j.2044-8260.2010.02006.x

[28] Rellini AH Vujanovic AA Gilbert M Childhood maltreatment and difficulties in emotion regulation: associations with sexual and relationship satisfaction among young adult women. J Sex Res 2012;49:434 42. 21512946 10.1080/00224499.2011.565430

[29] Kinzl JF Traweger C Biebl W Sexual dysfunctions: relationship to childhood sexual abuse and early family experiences in a nonclinical sample. Child Abuse Negl 1995;19:785 92. 7583734 10.1016/0145-2134(95)00048-d

[30] Maniglio R The role of parent-child bonding, attachment, and interpersonal problems in the development of deviant sexual fantasies in sexual offenders. Trauma Violence Abuse 2012;13:83 96. 22467644 10.1177/1524838012440337

[31] Zadra AL Nielsen TA Typical dreams: a comparison of 1958 versus 1996 student samples. Sleep Res 1997;26:280.

[32] Bernstein DM Roberts B Assessing dreams through self-report questionnaires: relations with past research and personality. Dreaming 1995;5:13 27.

[33] Chen W Qin K Su W Development of a structure-validated Sexual Dream Experience Questionnaire (SDEQ) in Chinese university students. Compr Psychiatry 2015;56:245 51. 25458478 10.1016/j.comppsych.2014.10.010

[34] Parker G Tupling H Brown LB A parental bonding instrument. Br J Med Psychol 1979;52:1 0.

[35] Perris C Jacobsson L Lindstrom H Development of a new inventory assessing memories of parental rearing behaviour. Acta Psychiatr Scand 1980;61:265 74. 7446184 10.1111/j.1600-0447.1980.tb00581.x

[36] Chen L Xu K Fu L Development of a structure-validated Family Relationship Questionnaire (FRQ) with Chinese university students. Bull Menninger Clin 2015;79:232 54. 26366981 10.1521/bumc.2015.79.3.232

[37] American Psychiatric Association. Diagnostic and Statistical Manual of Mental Disorder. 5th ed. Arlington, VA: American Psychiatric Association; 2013.

[38] Mathes J Schredl M Göritz AS Frequency of typical dream themes in most recent dreams: an online study. Dreaming 2014;24:57 66.

[39] Schredl M Continuity between waking and dreaming: a proposal for a mathematical model. Sleep Hypn 2003;5:38 52.

[40] Younis I Abdelrahman SH Ibrahim A Sex dreams in married women: prevalence, frequency, content, and drives. Dreaming 2017;27:251 9.

[41] Brotto LA Woo JS Gorzalka BB Differences in sexual guilt and desire in East Asian and Euro-Canadian men. J Sex Res 2012;49:594 602. 22004159 10.1080/00224499.2011.618956

[42] Woo JS Brotto LA Gorzalka BB The role of sex guilt in the relationship between culture and women's sexual desire. Arch Sex Behav 2011;40:385 94. 20349208 10.1007/s10508-010-9609-0

[43] Cipolli C Bolzani R Comoldi C Bizarreness effect in dream recall. Sleep 1993;16:163 70. 8446837 10.1093/sleep/16.2.163

[44] McNamara P Andresen J Clark J Impact of attachment styles on dream recall and dream content: a test of the attachment hypothesis of REM sleep. J Sleep Res 2001;10:117 27. 11422726 10.1046/j.1365-2869.2001.00244.x

[45] Yoon S Bellamy JL Kim W Father involvement and behavior problems among preadolescents at risk of maltreatment. J Child Fam Stud 2018;27:494 504. 29491703 10.1007/s10826-017-0890-6 PMC5826550

[46] Soffer-Dudek N Sadeh A Dream recall frequency and unusual dream experiences in early adolescence: longitudinal links to behavior problems. J Res Adolesc 2013;23:635 51.

[47] Manu AA Mba CJ Asare GQ Parent-child communication about sexual and reproductive health: evidence from the Brong Ahafo region, Ghana. Reprod Health 2015;12:16. 25889521 10.1186/s12978-015-0003-1 PMC4359389

[48] Karofsky PS Zeng L Kosorok MR Relationship between adolescent-parental communication and initiation of first intercourse by adolescents. J Adolesc Health 2001;28:41 5. 11137905 10.1016/s1054-139x(00)00156-7

[49] Kröner-Borowik T Gosch S Hansen K The effects of suppressing intrusive thoughts on dream content, dream distress and psychological parameters. J Sleep Res 2013;22:600 4. 23679926 10.1111/jsr.12058

[50] Seehuus M Clifton J Rellini AH The role of family environment and multiple forms of childhood abuse in the shaping of sexual function and satisfaction in women. Arch Sex Behav 2015;44:1595 608. 25339521 10.1007/s10508-014-0364-5

[51] Luebbe AM Bump KA Fussner LM Perceived maternal and paternal psychological control: relations to adolescent anxiety through deficits in emotion regulation. Child Psychiatry Hum Dev 2014;45:565 76. 24306144 10.1007/s10578-013-0425-3

[52] Sikka P Pesonen H Revonsuo A Peace of mind and anxiety in the waking state are related to the affective content of dreams. Sci Rep 2018;8:12762. 30143673 10.1038/s41598-018-30721-1 PMC6109051

[53] Moretti MM Craig SG Maternal versus paternal physical and emotional abuse, affect regulation and risk for depression from adolescence to early adulthood. Child Abuse Negl 2013;37:4 13. 23253857 10.1016/j.chiabu.2012.09.015

[54] Brotto LA Atallah S Johnson-Agbakwu C Psychological and interpersonal dimensions of sexual function and dysfunction. J Sex Med 2016;13:538 71. 27045257 10.1016/j.jsxm.2016.01.019

[55] Rich CL Gidycz CA Warkentin JB Child and adolescent abuse and subsequent victimization: a prospective study. Child Abuse Negl 2005;29:1373 94. 16293305 10.1016/j.chiabu.2005.07.003

[56] Hu MH Huang GS Huang JL Clinical characteristic and risk factors of recurrent sexual abuse and delayed reported sexual abuse in childhood. Medicine 2018;97:e0236. 29620636 10.1097/MD.0000000000010236 PMC5902297

[57] Luo TYE Sexual abuse trauma among Chinese survivors. Child Abuse Negl 1998;22:1013 26. 9793724 10.1016/s0145-2134(98)00079-9

[58] Vaillancourt-Morel MP Godbout N Labadie C Avoidant and compulsive sexual behaviors in male and female survivors of childhood sexual abuse. Child Abuse Negl 2015;40:48 59. 25435106 10.1016/j.chiabu.2014.10.024

[59] de Graaf H Vanwesenbeeck I Woertman L Parental support and knowledge and adolescents’ sexual health: testing two mediational models in a national Dutch sample. J Youth Adolesc 2010;39:189 98. 20084564 10.1007/s10964-008-9387-3

